# Plasmon-Enhanced Fluorescence of Single Extracellular Vesicles Captured in Arrayed Aluminum Nanoholes

**DOI:** 10.1021/acsomega.4c05492

**Published:** 2024-12-18

**Authors:** Yupeng Yang, Prattakorn Metem, Mohammad Hadi Khaksaran, Siddharth Sourabh Sahu, Fredrik Stridfeldt, André Görgens, Shi-Li Zhang, Apurba Dev

**Affiliations:** † Division of Solid-State Electronics, Department of Electrical Engineering, The Ångström Laboratory, Uppsala University, SE-751 03 Uppsala, Sweden; ‡ Division of Applied Electrochemistry, Department of Chemical Engineering, KTH Royal Institute of Technology, SE-100 44 Stockholm, Sweden; § Bio-Opto-Nano Physics, Department of Applied Physics, School of Engineering Sciences, KTH Royal Institute of Technology, SE-100 44 Stockholm, Sweden; ∥ Department of Laboratory Medicine, Division of Biomolecular and Cellular Medicine, Karolinska Institutet, 171 77 Stockholm, Sweden; ⊥ Department of Cellular Therapy and Allogeneic Stem Cell Transplantation (CAST), Karolinska University Hospital Huddinge and Karolinska Comprehensive Cancer Center, 113 51 Stockholm, Sweden; # Institute for Transfusion Medicine, University Hospital Essen, University of Duisburg-Essen, 45147 Essen, Germany

## Abstract

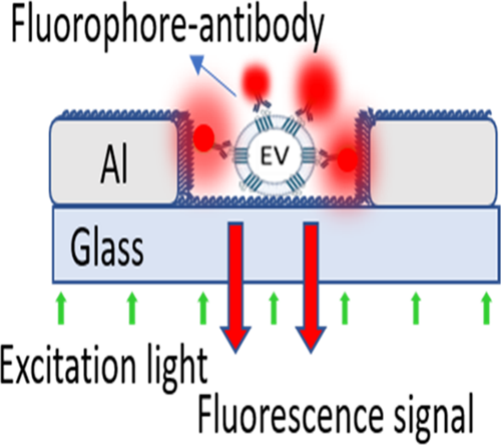

Extracellular vesicles (EVs) are nanoparticles encapsulated with a lipid bilayer, and they constitute an excellent source of biomarkers for multiple diseases. However, the heterogeneity in their molecular compositions constitutes a major challenge for their recognition and profiling, thereby limiting their application as an effective biomarker. A single-EV analysis technique is crucial to both the discovery and the detection of EV subpopulations that carry disease-specific signatures. Herein, a plasmonic nanohole array is designed for capturing single EVs and subsequently performing fluorescence detection of their membrane proteins by exploiting plasmonic amplification of the fluorescence signal. Unlike other reported methods, our design relies on an exclusive detection of single EVs captured inside nanoholes, thus allowing us to study only plasmonic effects and avoid other metal-induced phenomena while leveraging on the proximity of emitters to the plasmonic hotspots. The method is optimized through numerical simulations and verified by a combination of atomic force, scanning electron microscopy, and fluorescence microscopy. Fluorescence enhancement is then estimated by measuring the CD9 expression of small EVs derived from the human embryonic kidney (HEK293) cell line and carefully considering the spatial distribution of emission and excitation intensities. Fluorescence intensities of immunostained EVs show a moderate overall enhancement of intensity and follow the intensity trend predicted by simulation for nanohole arrays with different nanohole periods. Moreover, the number of observed EVs in the best-performing nanohole array increases by more than 12 times compared with EVs immobilized on a reference substrate, uncovering a vast amount of weakly fluorescent EVs that would remain undetected with the regular fluorescent method. Our nanohole array provides a basis for a future platform of single-EV analyses, also promising to capture the signature arising from low-expressing proteins.

## 1 Introduction

Extracellular vesicles (EVs) are a class of lipid-encapsulated nanovesicles that are secreted by most cell types in the human body. EVs carry a plethora of biological cargo, including proteins, lipids, and nucleic acids, from their parental cells. It has been discovered that EVs are instrumental in cell-to-cell communication and phenotype regulations.^1−5^ As such, research has been heavily geared toward the study of EVs during the past decades to understand their specific roles in intercellular communication and pathogenesis of diseases, including cancer and neurodegenerative disorders.^2,6−9^ These developments have significantly increased the demand to develop sensitive methods for EV analysis. However, EVs are both small and highly heterogeneous in their composition,^9,10^ thus rendering many of the established methods incompatible for practical usage.

In addition to their intraluminal cargo, EVs express a large variety of membrane proteins on their lipid bilayers. The composition of these membrane proteins has been reported to carry disease-specific and tissue-specific signatures and therefore has received wide attention.^1,11,12^ Specifically, expression levels of a common transmembrane protein family called tetraspanins, such as CD9, CD63, and CD81, have been widely studied,^4,13,14^ and thus profiling of these proteins has become a test bed to develop and validate new methods. Previously, we have demonstrated that the expression levels of these tetraspanins are highly heterogeneous.^9,10^ Several techniques have been employed to directly detect these tetraspanins and other membrane proteins; for instance, enzyme-linked immunosorbent assay (ELISA),^15^ Western blotting,^9^ and mass spectrometry.^16^ The fluorescence-based method has also been widely used as it allows for interrogating at a single-EV level and thus potentially revealing their compositional heterogeneity.^9,14,17−20^ Such heterogeneity, in turn, poses a major obstacle when profiling their membrane proteins, as EVs with a lower expression level of a target protein are prone to be left undetected during fluorescence analysis.^18,19^

To overcome such a drawback of conventional fluorescence techniques, plasmonic nanostructures have been recently explored to amplify the fluorescence signals of EVs.^18−25^ Localized surface plasmons (LSPs) and surface plasmon polaritons (SPPs) can strongly confine the electric field in the subwavelength volume. These strong electric fields can interact with fluorophores at the emission and excitation wavelengths, which can lead to enhanced absorption rate and quantum yield.^19,21^ Arrayed nanoholes in a metal film is one such plasmonic structure where the coupling wavelengths can be tuned through changes in the geometrical parameters, e.g., the diameter of the nanoholes, the period of the arrayed nanoholes, and the film thickness, thus, offering a major advantage.^26−29^ This simple approach has been explored for the detection of fluorescently tagged particles such as viruses, EVs, etc.,^18,29,30^ by randomly capturing the particles on such nanostructured metal films. However, given that the reflection from the metal film itself is known to strongly influence the fluorescence intensity,^31^ the analysis of plasmonic effects becomes complicated with such experimental designs. Besides, the localized plasmonic hotspots may induce nonuniform amplifications of the emitters thereby altering the distribution. A possible solution to such issues is to restrict the emitters within the nanoholes, so that the emitters remain within close proximity of plasmonic hotspots while avoiding reflection-induced effects.

With a selective capture of single quantum dots within metallic nanoholes, we have recently demonstrated such a prospect with an overall fluorescence enhancement of as much as 5-fold.^32^ In the present study, we have extended the concept to molecular profiling of EVs using an optimized nanoplasmonic design. To this end, we have simulated, optimized, and fabricated a functional chip with a plasmonic aluminum (Al) thin film as a potential platform for single-EV analysis. Small EVs (sEVs) derived from the human embryonic kidney (HEK293) cell line were successfully captured inside the nanoholes, and their CD9 expression was analyzed by using fluorescently tagged anti-CD9 antibodies. In our setup, the excitation light struck the chip from the glass side, and the emission fluorescence signals were collected from the same objective. By keeping the EV-bearing liquid on the metal side, it ensures that only the fluorescence signals of the EVs inside the nanoholes can be detected. With due considerations to the spatial distribution of the excitation intensity and heterogeneous CD9 expression, we estimated that under optimum conditions the fluorescent signals could be enhanced overall by 1.3-fold due to plasmonic effects. Our experimental results showed good agreement with the simulated data obtained from nanoholes with different periods. More importantly, we also observed a 12-fold increase in the density of the detected number of CD9-positive EVs captured inside the nanoholes as compared with that on a reference substrate.

## 2 Results and Discussion

### 2.1 Simulation and Fabrication of the Predesigned Nanohole Arrays in an Al Film

**Figure 1:**
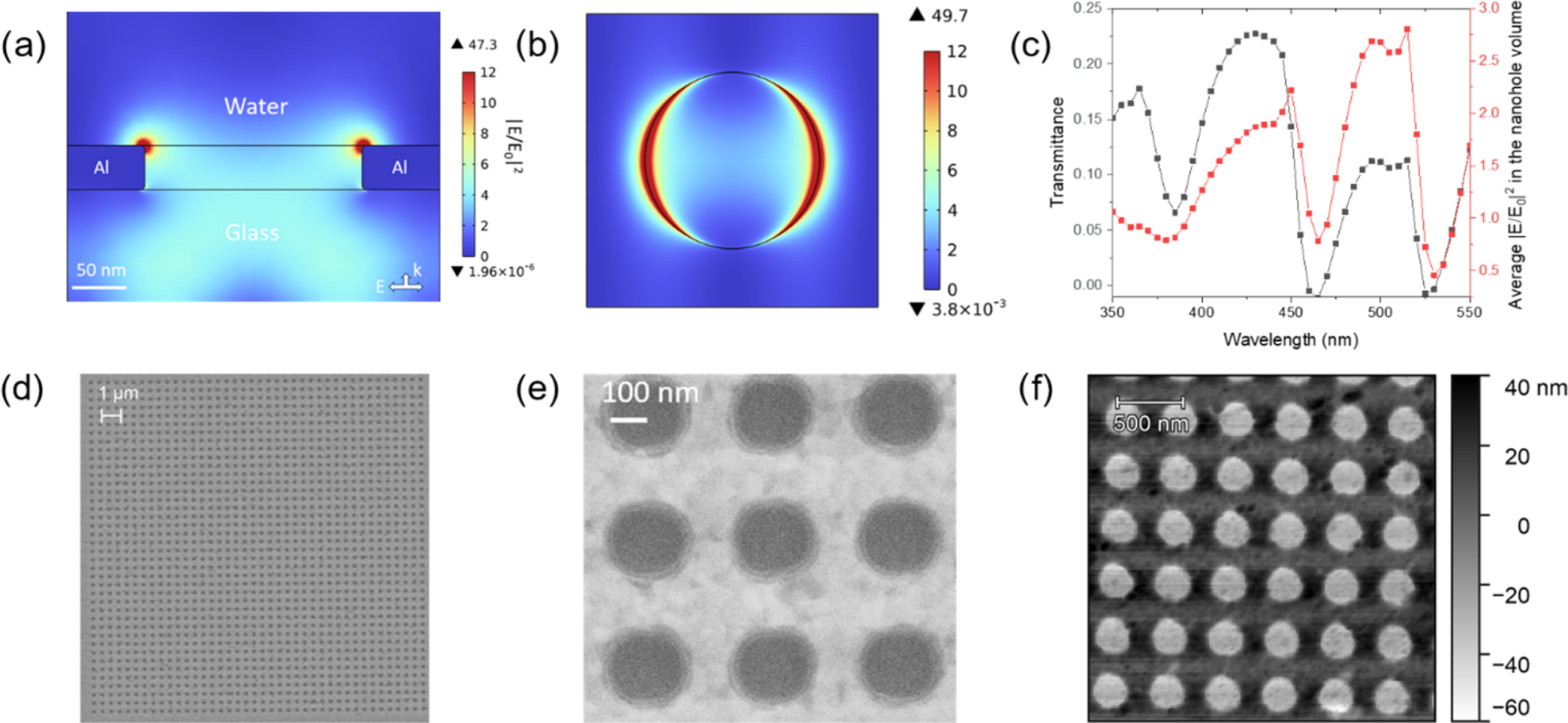
Simulated and fabricated nanohole arrays in an Al thin film. Simulated square of electric field enhancement, (*E*/*E*_0_)^2^, on the plane of the (a) central cross section and (b) top view of the Al top surface of a nanohole with a rounded rim at both top and bottom surface in an array under excitation at 515 nm wavelength (transmittance peak), *t =* 40 nm, *D =* 200 nm, and *p* = 340 nm. The color bar is on a linear scale. (c) Simulated transmittance spectrum of the nanohole array in panel (a) and the square of its average electric field enhancement in the nanohole volume. (d) SEM top-view image of a fabricated nanohole array on glass with a (e) zoom-in image. (f) AFM image of a fabricated nanohole array, with the Al thickness around 40 nm, as designed.

Aluminum was used for the design as it has many advantages compared to conventional noble materials such as Au and Ag. These include low cost, high natural abundance, high stability, CMOS compatible processing, broad plasmon resonance across the ultraviolet–visible-infrared wavelength region, and good adhesion of Al films to diverse substrates.^33,34^ Therefore, plasmonic nanohole arrays in a thin Al film on a glass substrate can be fabricated using sputtering, lithography, and dry-etching with good stability and at a relatively low cost. It is well-known that the plasmon-enhanced fluorescence is usually proportional to the square of the local electric field enhancement,^21,32^ which again depends on the geometrical parameters including period (*p*, e.g., for the periodical arrangement of nanoholes), thickness of the metal film (*t*), and diameter of nanoholes in the array (*D*).^32,35−37^ In order to find the optimized parameters, COMSOL simulation was performed, aiming to achieve the highest average electric field in the nanohole volume at the excitation wavelength of 515 nm. For the simulation, we considered a nanohole of 200 nm diameter formed in the Al film. As the fabrication of nanoholes with sharp-edge is difficult,^32^ we simulated the nanoholes with a rounded rim at both the top and bottom surface. The diameter of 200 nm was selected to allow capturing of single sEVs (sizes being smaller than 200 nm in diameter; see the nanoparticle tracking analysis result in Figure S1), while prohibiting multiple EVs in one nanohole. The simulation results performed for a nanohole array design with *p =* 340 nm, *t =* 40 nm, and *D =* 200 nm are displayed in Figure 1a,b. As seen, the electric field is more concentrated at the edges of the nanoholes and is parallel to the electric field polarization direction of the incident light. The average electric field enhancement in the nanohole volume agrees well with the transmittance spectrum of the nanohole array, presented in Figure 1c, i.e., the transmittance increase with the average electric field in the nanohole volume,. The dependence of the average electric field amplification factor (*E*/*E*_0_) on the nanohole period and Al thickness as a function of wavelength (350 to 550 nm) was also investigated, as shown in Figure S2d,e. The general trend of the amplification factor remains similar for the different periods except that the spectrum red-shifts with increasing period. However, the behavior is almost independent of the film thickness. The observations agree with the previously reported dependence of interference of surface plasmon polariton mode on the periodicity of the nanohole array.^34,38,39^ Guided by the simulation, three nanohole arrays with different nanohole periods *p* = 305, 340, and 430 nm were fabricated on the same chip with the same film thickness (*t =* 40 nm) and nanohole diameter (*D =* 200 nm). Fabrication of the designed nanohole arrays was done by multiple optimized processing steps including sputter-deposition of the Al film, electron-beam lithography (EBL), and inductively coupled reactive-ion etching (ICP-RIE), as reported in our previous study.^32^

Circular holes with a diameter around 200 nm were successfully obtained after optimizing the processing steps (Figure S3 supported by a detailed description in “Section 6”), as shown by a representative scanning electron microscopy (SEM) image presented in Figure 1d. The magnified image presented in Figure 1e shows uniform and nearly circular openings in the Al film. Atomic force microscopy (AFM) was then utilized for high-resolution imaging of the nanohole array in order to measure the depth profile (see Figure 1f). The depth of the nanoholes was measured to be 44.4 ± 2.9 nm, close to the designed value of 40 nm. The slight deviation from the desired depth and the depth variations among the holes are likely due to the inevitable fabrication variations including film thickness, etch of the underlying glass (the so-called overetch), and etch nonuniformity. These results substantiate that the nanohole arrays could be successfully fabricated following the intended design parameters.

### 2.2 Optimized Capturing and Immunostaining of EVs

**Figure 2:**
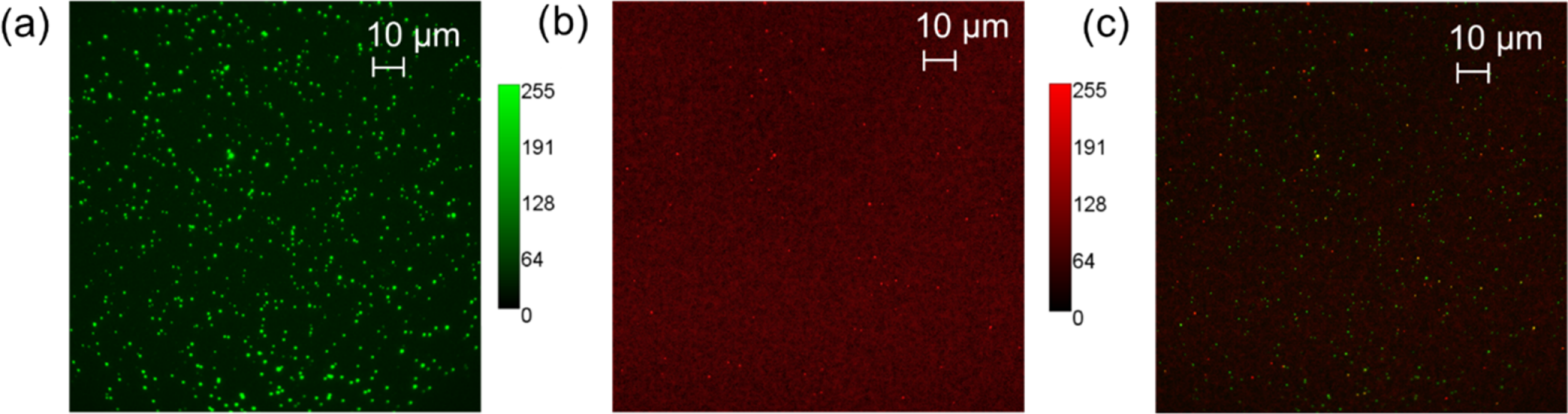
Fluorescence images of mNG-EVs immunostained with R-PE-anti-CD9 captured on the reference substrate (a) in the mNG channel, (b) in the R-PE channel, and (c) as a combined image.

Before proceeding with the plasmonic nanohole arrays, the experimental parameters, such as the density of captured EVs and their immunostaining protocol, were optimized. Bioengineered EVs derived from the human embryonic kidney (HEK293) cell line and tagged with mNeonGreen fluorescent protein (referred to as mNG-EVs hereafter) fused to CD63 were used for this purpose. The selection of mNG-EVs for this step was for the sake of simplifying the experimental design for this part as the bright fluorescent proteins expressed in the EVs can be easily imaged without requiring further immunostaining steps. In order to estimate the density of the captured EVs, the mNG-EVs were captured on a poly(l-lysine) (PLL) functionalized bare glass substrate and analyzed with a wide-field inverted epi-fluorescence microscope under light-emitting diode (LED) excitation having its center wavelength at 475 nm. A representative fluorescence image of the substrate after being incubated with 25 μL of 2.0 × 10^9^ EVs/mL mNG-EVs in phosphate-buffered saline (PBS) solution is shown in Figure 2a. The density of fluorescence spots increases, as expected, with the concentration of EVs (according to the concentration dependent study presented in Figure S4a). The concentration of 2.0 × 10^9^ EVs/mL provides the optimum surface density of the captured EVs, still having an average distance between neighboring EVs large enough to be separately identified. Hence, the concentration of 2.0 × 10^9^ EVs/mL was utilized for subsequent experiments.

Next, we investigated the immunostaining protocol. For this purpose, 4 nM of R-PE conjugated anti-CD-9 antibody was used to stain the captured mNG-EVs. The corresponding fluorescence images are shown in Figure 2b. The overlapping image, i.e., the green channel (mNG fluorescence signal) and the red channel (R-PE fluorescence signal), is presented in Figure 2c. The yellow fluorescence spots in Figure 2c confirm the successful staining of mNG-EVs with the R-PE labeled anti-CD9 antibody. The Venn diagram presented in Figure S4c shows that only about 8% (67 fluorescence spots out of the 784 spots) of all of the detected EVs show coexpression of CD9 with CD63. The spots with only R-PE signals could be related to EVs with an undetectable expression of the mNG signal and/or some nonspecific binding of the antibodies. There is almost no correlation between the mNG and R-PE signals, i.e., the CD63 protein and CD9 surface proteins on EVs (see Figure S4b for the scattering plot).

**Figure 3:**
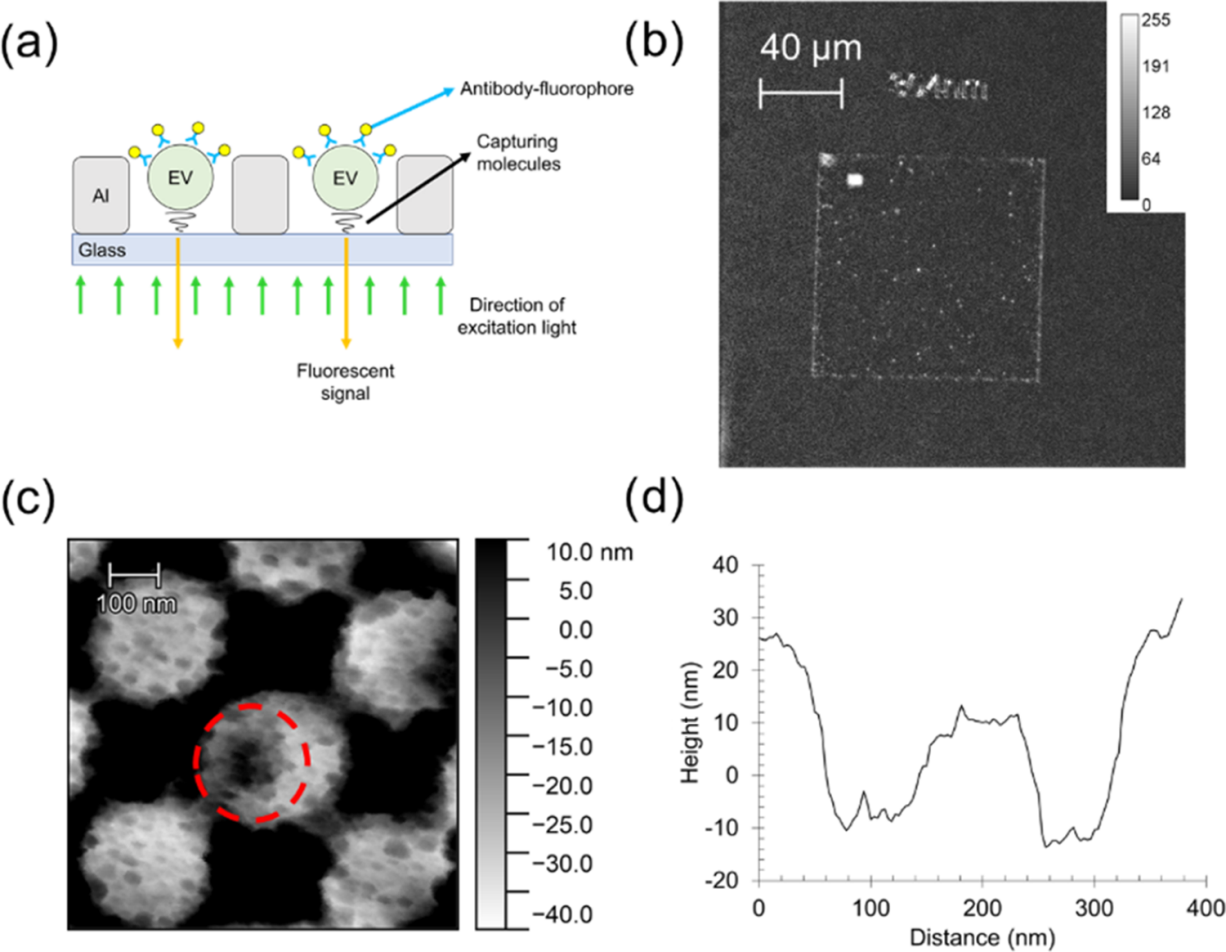
EVs captured in an optimized nanohole array. (a) Schematic of a nanohole array with EVs captured inside the holes during fluorescent analysis with both the excitation light and the emission fluorescent signals collected from the glass side. (b) Dark-field fluorescence image of R-PE stained wt-EVs inside a nanohole array under 515 nm laser excitation. (c) AFM image of a single wt-EV inside a nanohole with height difference shown in panel (d).

Following our protocol above, the capturing and immunostaining method was verified using a fabricated nanohole array. As before, an LED source coupled with an epi-fluorescence microscope was used. It was first confirmed that mNG-EVs could also be successfully captured in nanohole arrays (100 × 100 nanoholes) by PLL (cf. a representative wide-field image in Figure S5 of mNG-EVs taken at a 475 nm LED excitation). There was no fluorescent signal in the control sample with an identical nanohole array but without EV incubation. Since the fluorescence signal was collected from the glass side using an inverted microscope, the mNG-EVs were surely lying inside the nanoholes. It should be noted that the number density of fluorescence spots detected from the nanohole array (Figure S5b) is a bit lower than that observed with bare glass substrate (Figure 2a). This is because the metal layer effectively blocks a majority of them unless they are inside the nanoholes, and there is no plasmonic effect for mNG-EVs with 475 nm LED excitation. Given the very low abundance of CD9-positive EVs in this sample (see Figure 2d), generating enough counts in nanohole arrays is difficult with immunostained antibody. Therefore, wild-type HEK293 EVs (wt-EVs) that contain a much larger proportion of CD9-positive EVs (Figure S6) were used to investigate the plasmonic effect of the nanohole array. In addition, a larger array (100 μm × 100 μm with 900 × 900 nanoholes) was adopted to increase the number of detected EVs. Furthermore, a monochromatic laser source was employed to match the simulated parameters. A schematic of the experimental design is depicted in Figure 3a. An inverted epi-fluorescence microscope equipped with a 515 nm laser was used to measure the plasmon-enhanced fluorescence signals from the EVs. A dark-field fluorescence image of EVs in one nanohole array is shown in Figure 3b, clearly visualizing single fluorescence spots arising from the immunostained EVs (anti-CD9-R-PE) inside the nanoholes. Control images taken on an identically prepared nanohole array but without any EVs and anti-CD9-R-PE did not show distinct fluorescent spots, as expected, except for fluorescent signals from the edge of the nanohole array, likely due to light scattering (Figure S7). The EV capture in the PLL-functionalized nanohole array was also confirmed by using AFM, as presented in Figure 3c. The AFM micrograph shows a single EV inside one nanohole as depicted with a red dashed circle. The height profile was extracted, as shown in Figure 3d, indicating that the captured EV is approximately 30 nm in height and roughly 100 nm in diameter. The flattening of surface-adhered EVs (width > height) is a common observation when being profiled using AFM.^8,40,41^ However, given the sparse distribution of EVs in the array (see Figure 3b), it was difficult to obtain more such AFM micrographs. In brief, the results presented above verify the capture of single EVs in the PLL-functionalized nanohole array.

### 2.3 Plasmon-Enhanced Fluorescence from EVs in the Nanoholes

**Figure 4:**
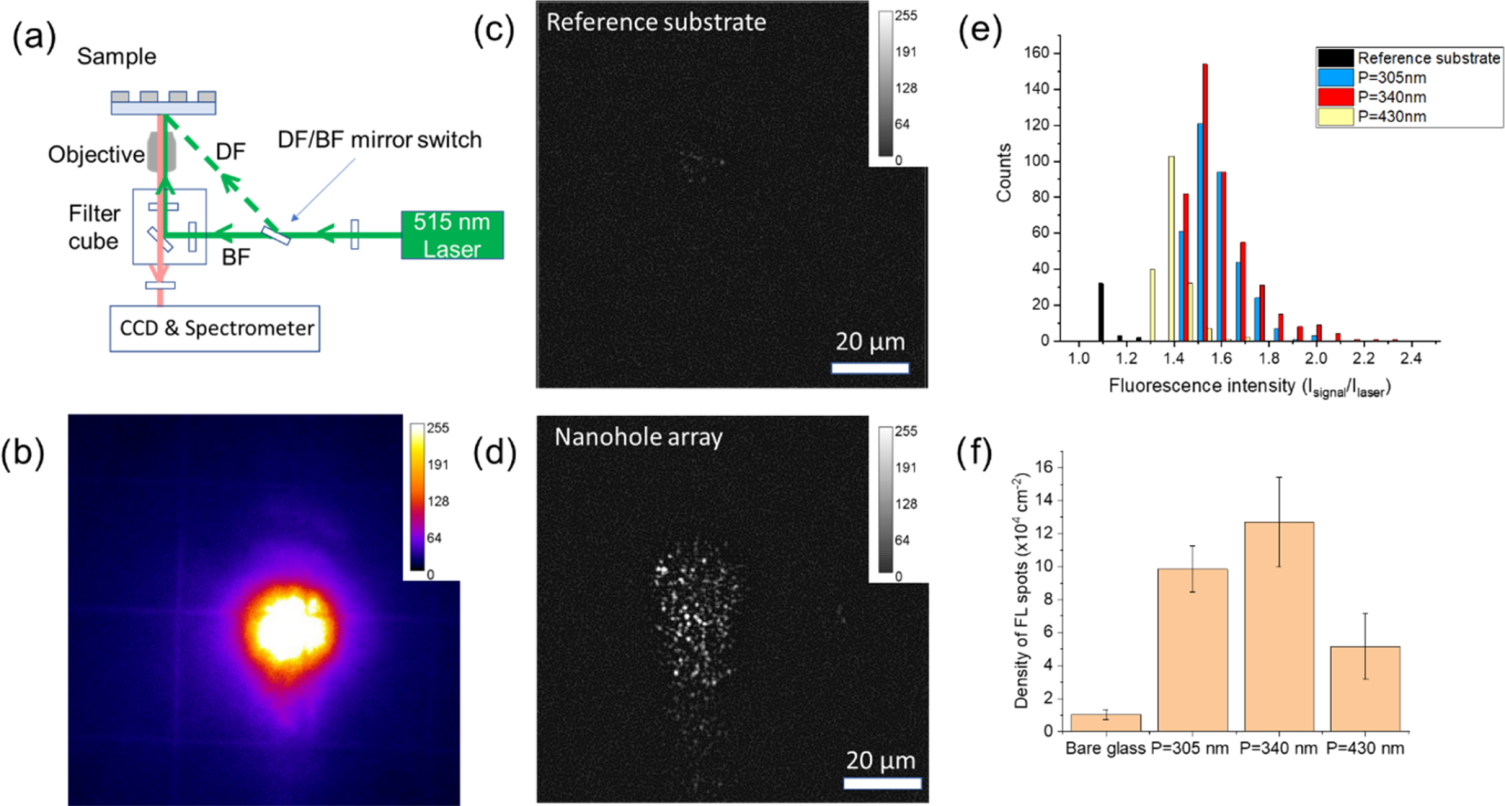
Bright-field fluorescence imaging and statistical analysis of R-PE stained EVs in multiple nanohole arrays with different nanohole periods and on a bare glass substrate as reference. (a) Schematic of the μPL system with bright-field and dark-field imaging modes. (b) Intensity profile of the focused laser spot on the substrate in the bright-field imaging mode, view size 100 μm × 100 μm. (c) A typical bright-field fluorescence image of R-PE stained EVs on a bare glass substrate and inside the nanoholes (d) under the excitation of 515 nm wavelength after background subtraction. (e) Histogram showing the fluorescence intensity distribution of EVs in nanohole arrays of *p* = 305, 340, and 430 nm, all with *t* = 30 nm and *D* = 200 nm. The distribution obtained from a reference substrate (bare glass) is also presented. (f) Histogram of the density of detected FL spots in these three different nanohole arrays and on the reference substrate.

While the dark-field images can be useful to excite larger areas, thereby generating larger counts, the oblique incidence of the exciting laser source makes the imaging condition different from that used for simulation where a perpendicularly incident light path was assumed. Thus, for a quantitative analysis of the fluorescence enhancement, bright-field imaging was employed (see Figure 4a). Since the incident laser intensity is usually inhomogeneous, as Figure 4b shows, the ratio of fluorescence signal to local laser spot intensity (*I*_signal_/*I*_laser_) was first calculated for different pixels in order to facilitate an accurate quantification of fluorescence enhancement due to plasmonic effects (see Figure S8). Representative images of single EVs captured on a bare glass substrate and in a nanohole array within the incident laser spot with an exposure time of 1 s are presented in Figure 4c,d. To maintain identical conditions, a control substrate after removal of the deposited Al film on the same chip was used as a reference and imaged with the same imaging settings. The reference substrate and nanohole arrays had the same surface functionalization and were incubated in the same solution for capturing wt-EVs. The distribution of *I*_signal_/*I*_laser_ presented as histograms for each nanohole period is shown in Figure 4e. The density of the fluorescence spots is also plotted as a histogram in Figure 4f. It is clear that both the overall fluorescence intensity and the detected counts of EVs are larger in the nanohole arrays compared with those on the reference substrate. The other parameters, including the mode of fluorescence intensity, overall fluorescence enhancement factor, and simulated volumetric |*E*/*E*_0_|^2^, are also summarized in Table 1. It can be seen that the nanohole array with a period of 305 nm exhibits the highest fluorescence enhancement factor of 1.35 among the investigated arrays. A comparison between the brightest EVs on the reference substrate and those in the nanohole arrays suggests that some EVs have been amplified by more than a factor of 2. The experimental values for different nanohole periods agree qualitatively with the trend predicted by the simulation. Along with the enhanced excitation rate, the quantum yield of R-PE fluorophores may also be increased due to the Purcell effect. The transmittance peaks of the nanohole array also overlap with the emission wavelength of the R-PE fluorophore (550–650 nm). Interestingly, a 12-fold increase in the EV density was observed in the nanohole array compared to that on the reference substrate despite a low average fluorescence enhancement factor. The density of detected EVs in a nanohole array was calculated after calibrating the area ratio of a nanohole to a unit cell of an array with a specific period. Further analysis of the histogram (presented in Figure S9) clearly shows that the increase in the density was observed almost entirely in the low-intensity part, clearly indicating that it is the low CD9-expressing EVs that primarily contribute to the increase in the number density.

**Table 1:** Statistical Analysis of the Fluorescence of EVs in Several Nanohole Arrays with Reference to that on a Bare Glass Substrate

period of nanohole arrays (nm)	density of detected EVs (×10^4^/cm^2^)	mode of fluorescence intensity (a.u.)	overall fluorescence enhancement factor	simulated volumetric average |*E*/*E*_0_|^2^
reference substrate	1.03	1.12	1.00	
305	9.85	1.51	1.35	3.84
340	12.7	1.49	1.33	2.79
430	5.15	1.32	1.18	2.34

The heterogeneity of protein expression on the EV-membrane has been widely studied and low-expressing EVs are known to dominate the distribution.^10^ While it is generally anticipated that a population of such low-expressing EVs may remain undetected in most fluorescent methods, the topic so far has not received much attention except for a recent study involving a similar plasmonic design.^18^ Indeed, the identification and study of such low-expression EVs may have important biological and pathological implications. However, plasmonic-based amplification particularly when metallic nanoholes are used has its own caveats and requires some careful evaluation of the experimental designs/parameters, which is the main focus of the present study. First, the excitation of fluorophores on a reflective substrate, e.g., with a metal coating, can induce a strong amplification due to the modification of both excitation and emission processes, as well as emission lobes.^31^ A 30-fold fluorescence enhancement of a cyanine 3 dye layer and a 4-fold mean signal amplification of homogeneously stained micrometer-sized objects was reported simply by using such a reflective surface.^31^ Clearly, in the backdrop of such a strong effect, it is challenging to estimate the plasmon-induced amplification. In our study here, this issue is addressed by analyzing EVs exclusively lying inside nanoholes, i.e., metal-free regions, through an appropriate experimental design. Although EVs were also found on the top surface of the Al film, they were not detected due to the opaque nature of the Al film (Figure S10 and Table S1).

Second, an accurate estimation of fluorescence enhancement is challenging due to heterogeneous distribution of proteins on the EV-membrane and variation of the location of EVs inside nanoholes, thus resulting in a broad distribution of fluorescence intensities from single EVs. This is further complicated by the spatial distribution of the excitation density (see Figures 2a and 4a) as the emitter lying in different locations within the Gaussian-like laser spot can experience different excitation intensities. The inhomogeneous laser spot issue is addressed by carefully estimating the excitation and emission intensities at individual pixels and normalizing them for a fair comparison. Finally, the experimental and simulation parameters were carefully selected and optimized to reduce uncertainties that may influence the estimated amplification factor. This effort includes a reference substrate that resembles the condition inside the nanohole array, rounded-rim nanoholes in simulation that resemble the fabricated nanoholes, and identical light path and monochromatic excitation source in both the experiment and simulation. The results provide a more reliable estimation, and the method reported here will guide future developments of plasmonic sensors. In future studies, it would be interesting and meaningful to investigate the plasmon-enhanced multiplexed profiling of EVs in nanoholes.

In conclusion, a plasmonic-based method to amplify fluorescence-based EV detection is reported. Plasmonic nanohole arrays in a thin Al film were used to enhance the fluorescence signals from the EVs sitting only inside the nanoholes. The nanohole array is first designed and optimized by simulation to have a maximum volumetric average electric field in the nanohole at the excitation wavelength. Then, the designed nanohole arrays are successfully fabricated on a glass substrate. Bioengineered mNG-EVs are used first for optimization of capture and immunostaining using both AFM and fluorescence imaging. The wt-EVs with more CD-9 on the surface and tagged by anti-CD9-R-PE are used to investigate the plasmonic effect of the nanohole array. The overall fluorescence intensity shows a 1.3-fold enhancement compared to that on the reference substrate. Moreover, the number of observed EVs in the best-performing nanohole array increases by more than 12 times compared to EVs immobilized outside of the nanostructure, uncovering a vast amount of weekly fluorescent EVs that would remain undetected by the regular fluorescent method. Therefore, this work provides a basis for highly sensitive and unbiased single-EV fluorescence detection and analysis.

## 3 Materials and Reagents

Phosphate buffer saline (PBS) in tablet form, poly-l-lysine (PLL), streptavidin, and casein (C5890) in powder form were obtained from Sigma-Aldrich. mNG-EV and wt-EVs were received through collaboration with Department of Laboratory Medicine, Division of Biomolecular and Cellular Medicine, Karolinska Institute, at two different concentrations of approximately 1 × 10^10^ and 1.4 × 10^11^ particles per mL. R-phycoerythrin (R-PE) conjugated anti-human CD9 was purchased from EXBIO, Czech Republic (clone MEM-61, 1P-208-T025).

HEK293T (human embryonic kidney-293T) cells were propagated in Dulbecco’s modified Eagle’s medium (DMEM) containing Glutamax-I and sodium pyruvate (4.5 g/L Glucose; Invitrogen) supplemented with 10% fetal bovine serum (FBS; Invitrogen) and 1% Antibiotic-Antimycotic (Anti-Anti; ThermoFisher Scientific). Cells were washed with PBS 48 h prior to collection of conditioned media for EV isolation from HEK293T cells, and the medium was changed to OptiMem (Invitrogen). HEK293 Freestyle suspension cells (HEK293FS; ThermoFisher Scientific) were cultured in chemically defined FreeStyle 293 Expression Medium (ThermoFisher Scientific) in 125 mL polycarbonate Erlenmeyer flasks (Corning) in a shaking incubator (Infors HT Minitron) according to the manufacturer’s instructions.^42^ All cell lines were grown at 37 °C, with 5% CO_2_ in a humidified atmosphere, and regularly tested for the presence of mycoplasma. The creation and characterization of the genetically modified stable cell line HEK293FS:CD63 mNG (HEK293 FreeStyle:CD63 mNeonGreen) was described previously.^10^

## 4 EV Preparation and Nanoparticle Tracking Analysis

Cell culture-derived conditioned media (CM) was first precleared from cells and debris by low-speed centrifugation at 700*g* for 5 min. A subsequent centrifugation at 2000*g* for 20 min would remove larger particles and debris. Next, the medium was filtered through 0.22 μm bottle top vacuum filters (Corning, cellulose acetate, low protein binding) to remove any larger particles. Precleared CM was subsequently concentrated via tangential flow filtration (TFF) by using the KR2i TFF system (SpectrumLabs) equipped with modified poly(ether sulfone) (mPES) hollow fiber filters with 300 kDa membrane pore size (MidiKros, 370 cm^2^ surface area, SpectrumLabs), at a flow rate of 100 mL/min (transmembrane pressure at 3.0 psi and shear rate at 3700 s^–1^), as described previously.^43,44^ Amicon Ultra-0.5 10 kDa MWCO spin-filters (Millipore) were used to concentrate the sample to a final volume of ∼100 μL. EVs were stored in Maxymum Recovery polypropylene 1.5 mL tubes (Axygen Maxymum Recovery, Corning, cat MCT-150-L-C) in PBS-HAT buffer (PBS, 25 mM Trehalose, 25 mM HEPES, 0.2% Human Serum Albumin) before usage as described previously.^45^ Nanoparticle tracking analysis (NTA)^46^ was applied to determine particle size and concentration of all samples using the NanoSight NS500 instrument equipped with NTA 2.3 analytical software and an additional 488 nm laser. The samples were diluted in 0.22 μm of filtered PBS to an appropriate concentration before being analyzed. At least five 30 s videos were recorded per sample in light scatter mode with a camera level of 11–13. Software settings were kept constant for all EV measurements; the analysis was performed with the screen gain at 10 and the detection threshold at 7 for all EV measurements.

## 5 Design and Simulation of Nanohole Array

The schematic of the simulation model is shown in Figure S2. Aiming to capture only single sEVs (50–200 nm in diameter) in the nanoholes, the diameter of the nanohole was designed to be 200 nm. The nanoholes were spread out as a square array separated by a period of 300 nm or larger. Aluminum (Al) was selected as a material of choice for the fabrication of the nanostructured thin film to accommodate analyses in the visible wavelength region.^34^ Thickness of the Al film was chosen to be in the range of 30–50 nm. The finite element method (FEM) based commercial software COMSOL Multiphysics 6.0 was used to find the optimum values of the thickness and periodicity of the nanohole array to achieve the highest local electric field enhancement at the excitation wavelength for R-PE, which has an absorption peak at 515 nm. The geometry of the nanohole for the simulation is shown in Figure S2. As shown, an Al thin film on the glass substrate was selected with water as a medium to reflect the actual measurement conditions. Considering the symmetry of the nanohole, only a quarter of the nanohole with 200 nm diameter and a 5 nm radius-shaved off edge was constructed and used in the simulation in order to save calculation time. To simulate the repetition of holes in the nanohole array, perfect electric and magnetic field boundary conditions were implemented. Excitation wavelengths from 350 to 550 nm were chosen with the light path from the glass to the metal nanohole array to represent the experimental setup. As the electric field of the incident light was set to 1 V/m, the volumetric average of the electric field norm inside the nanohole was considered to directly represent the amplification of the electric field in the nanohole. The average electric field norm in the nanohole at each wavelength was calculated while geometrical parameters were adjusted to tune the amplification maximum at the desired wavelength of 515 nm.

## 6 Nanohole Array Fabrication

The fabrication procedure of the Al nanohole array has been previously reported by our group^32^ and a flowchart is schematically shown in Figure S3. Briefly, a glass coverslip with a thickness of 170 μm was cleaned by following the RCA standard cleaning procedure. The coverslip was further cleaned using oxygen plasma (Tepla 300) prior to the sputter-deposition of Al films (von Ardenne CS730S) to predefined thicknesses. Subsequently, the Al surface was spin-coated by the ARP 6200.09 electron-beam resist (Allresist GmbH) and the nanohole array pattern was drawn by means of EBL (nB5, NanoBeam, U.K.). After development in the AR 600–546 developer (Allresist GmbH), the nanohole pattern was transferred into the Al film by means of inductively coupled plasma-reactive-ion etching (ICP-RIE, PlasmaTherm SLR). The residue resist was then completely removed using the AR 600–71 resist remover (Allresist GmbH), rinsed with DI water, and blown dry. The nanohole arrays were characterized using scanning electron microscopy (SEM, LEO 1530, Zeiss) and atomic force microscopy (AFM, NanoWizard 3, JPK Instruments).

## 7 Optimization of EV Capturing and Immunostaining

To optimize the capturing and immunostaining procedure, bioengineered mNeonGren-tagged-CD63 HEK293 EVs (mNG-EVs) were used, due to their innate fluorescence from the green fluorescent protein mNeonGreen, on a 170 μm thick glass coverslip. The functionalization procedure is briefly summarized in Figure S11 and has been previously reported by our group.^9,14^ The substrate was rinsed with acetone, isopropanol (IPA), and DI water and dried using N_2_ gas. A silicone well (IBIDI Culture-Inserts) was attached to the substrate, in which a solution of 100 μg/mL poly-l-lysine (PLL) in filtered DI water was inserted and incubated for 5 min. After washing the substrate with phosphate buffer saline (PBS), EVs were incubated at room temperature for 1 h. For optimization, various concentrations of mNG-EVs in PBS (6.7 × 10^8^, 1 × 10^9^, 2 × 10^9^ EVs/mL) were used during the incubation. Subsequently, the substrate was washed thoroughly with PBS to remove any unattached EVs from the surface. The captured EVs were observed using a wide-field fluorescence microscopy (Colibri 5, ZEISS) with a 100× magnification oil immersion objective lens and a 475 nm LED as the excitation source. The number of fluorescence spots observed under the microscope was compared, and the optimal concentration of EVs was selected for the experiments with nanoholes. Before immunostaining, a casein solution at a concentration of 1 mg/mL was incubated overnight prior to the antibody incubation to prevent unspecific binding of antibodies. After that, anti-CD9 antibody conjugated with R-phycoerythrin (R-PE) at a concentration of 4 nM was incubated for 1 h. It was then washed thoroughly with 1× PBS. A 555 nm LED light source (ZEISS Colibri) was used for the excitation of the R-PE.

## 8 Characterization of EVs in the Nanoholes

To verify the capture of single EVs inside the nanoholes, both AFM and fluorescence microscopy were used. For this purpose, wt-EVs captured in the nanohole array were imaged using AFM in quantitative imaging mode. After the EVs were stained with an anti-CD9 R-PE conjugate, a μPL fluorescence microscope system (built on Zeiss Axio Observer Z1), with a 515 nm laser, a 63× air objective with window correction, and a 550 nm high-pass emission filter, was used for fluorescence imaging. Images were recorded using both dark-field and bright-field configurations. The intensities of the fluorescence spots under bright-field imaging mode were analyzed and compared with the R-PE stained EVs on the bare substrate region on the same chip.

## Supporting Information Available

Additional simulation settings and results; schematics of materials and experimental setups; and fluorescence measurement results (PDF)
