# Supplementary material for: Plasmon-Enhanced Fluorescence of Single Extracellular Vesicles Captured in Arrayed Aluminum Nanoholes
Source: ACS Omega. 2024 Dec 18;9(52):51022–30. doi: 10.1021/acsomega.4c05492 (PMC11696387; doi:10.1021/acsomega.4c05492)
Supplement: Supplementary file 1 — ao4c05492_si_001.pdf [file ao4c05492_si_001.pdf]

Supporting Information for

Plasmon-Enhanced Fluorescence of Single Extracellular Vesicles Captured in Arrayed Aluminum Nanoholes

Yupeng Yang,[□*] *Prattakorn Metem,*[†] *Mohammad Hadi Khaksaran,*[□] *Siddharth Sourabh Sahu,*[□]

Fredrik Stridfeldt,[Ʋ] *André Görgens,*[ϕ,ȷ,Φ] *Shi-Li Zhang,*[□] *Apurba Dev*[□,Ʋ,*]

[□]Division of Solid-State Electronics, Department of Electrical Engineering, The Ångström Laboratory, Uppsala University, SE-751 03 Uppsala, Sweden, [†]Division of Applied Electrochemistry, Department of Chemical Engineering, KTH Royal Institute of Technology, SE-100 44 Stockholm, Sweden, [Ʋ]Bio-Opto-Nano Physics, Department of Applied Physics, School of Engineering Sciences, KTH Royal Institute of Technology, SE-100 44 Stockholm, Sweden, [ϕ]Department of Laboratory Medicine, Division of Biomolecular and Cellular Medicine, Karolinska Institutet, Stockholm, Sweden, [ȷ]Department of Cellular Therapy and Allogeneic Stem Cell Transplantation (CAST), Karolinska University Hospital Huddinge and Karolinska

Comprehensive Cancer Center, Stockholm, Sweden, [Φ]Institute for Transfusion Medicine,

University Hospital Essen, University of Duisburg-Essen, Essen, Germany

[*]Email: yupeng.yang@angstrom.uu.se, apurba.dev@angstrom.uu.se

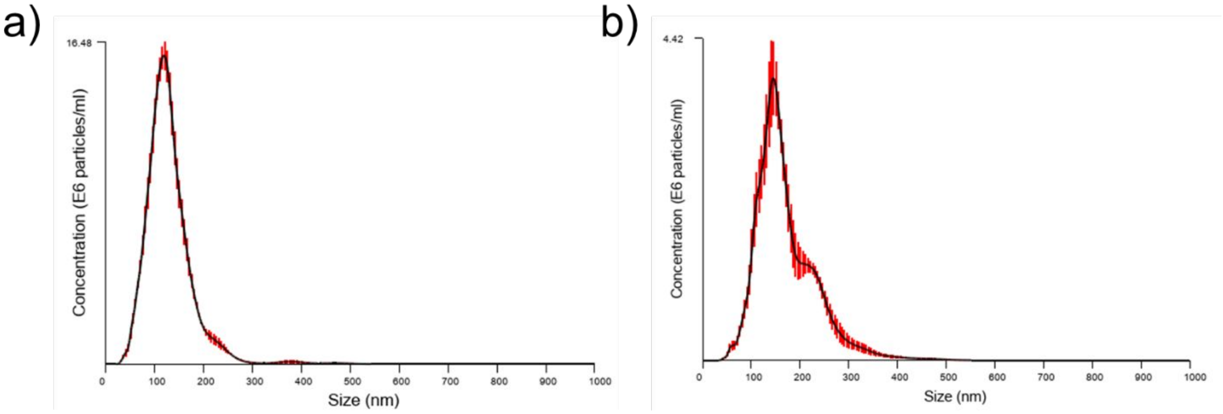

Figure S1. NTA results of mNG-EVs (a) and wt-EVs (b). Red error bars indicate +/- 1 standard error of the mean.

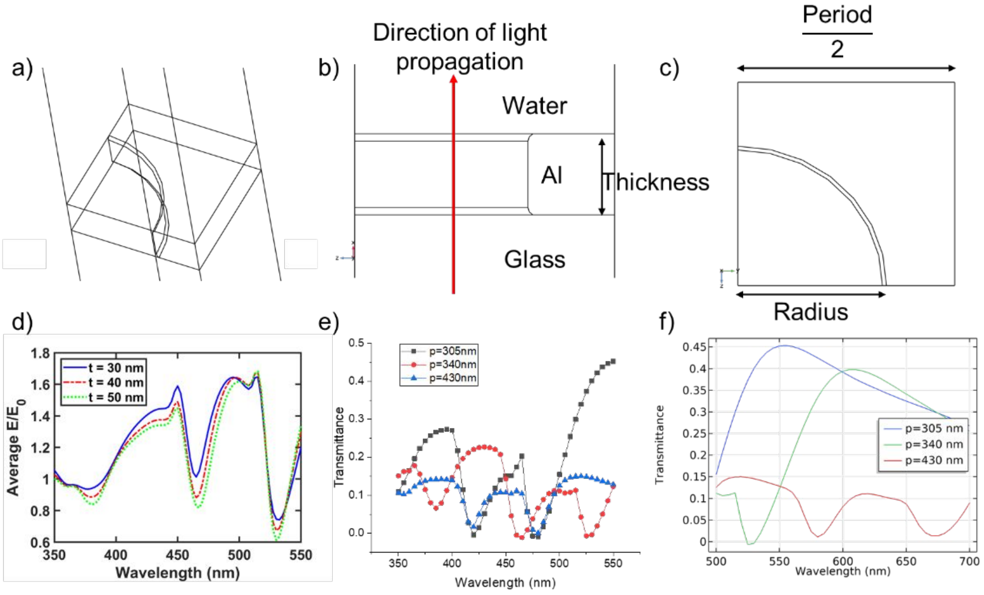

Figure S2. The main part of the model used in the simulation. (a) 3D view of a quarter of a cylindrical nanohole. (b) Side-view of half of the nanohole with the indication of light direction of propagation. (c) Top-view of a quarter of the nanohole. (d) Electric field enhancement spectrum with p=340 nm and D=200 nm. Simulated transmittance spectra of nanohole arrays with t=40 nm, D=200 nm, and different periods in the wavelength range from 350 nm to 550 nm (e) and from 500 nm to 700 nm (f).

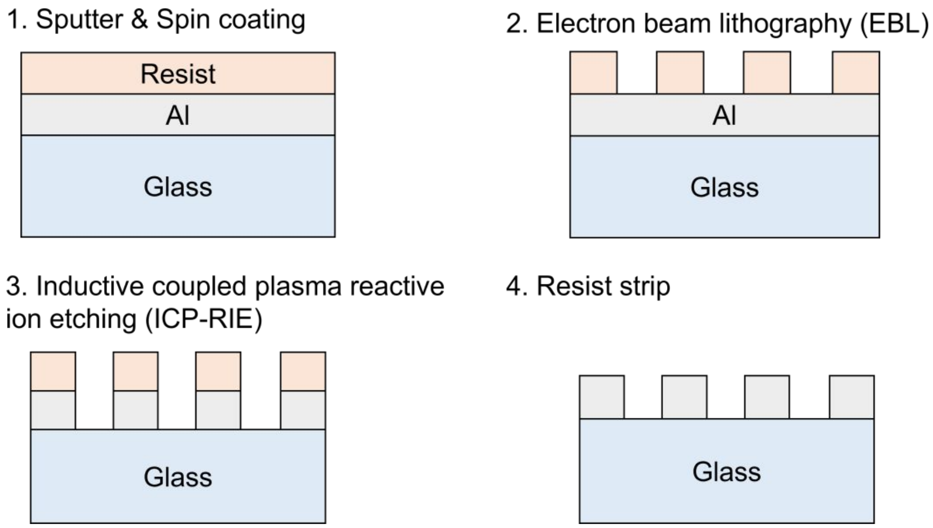

Figure S3. Schematic of the fabrication procedure for the nanohole array: 1. Al sputter-deposition and resist spin-coating, 2. patterning the resist using EBL, 3. with the patterned resist acting as a mask for inductive plasma reactive ion etching (ICP-RIE) to form nanoholes in the Al film, 4. resist removal followed by substrate cleaning.

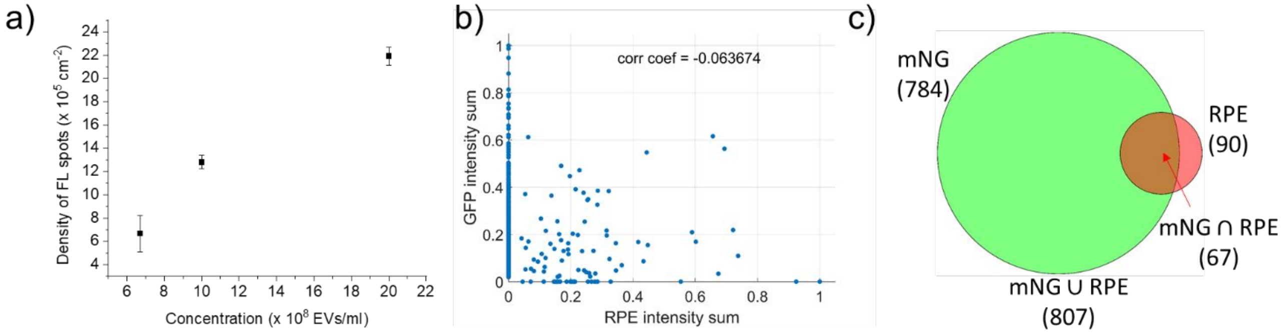

Figure S4. (a) Increased density of FL spots on a reference substrate with the concentration of EVs, as expected. (b) Statistically analyzed colocalization and correlation results from Figure 2a-c. (c) Colocalization analysis between the EVs observed in the mNG and R-PE channels showing the EV counts in each channel along with the number of EVs exhibiting colocalization.

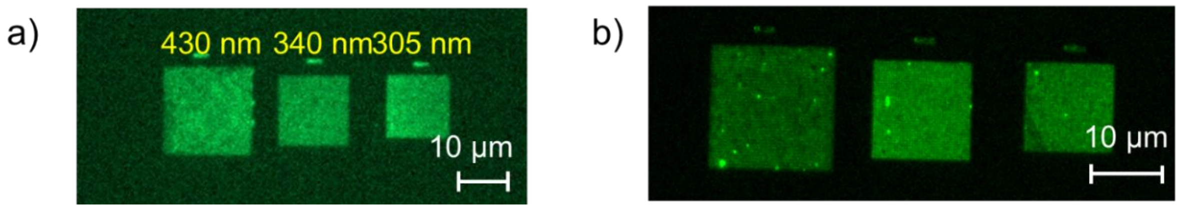

Figure S5. Fluorescence image of 3 nanohole arrays before (a) and after (b) capturing EVs.

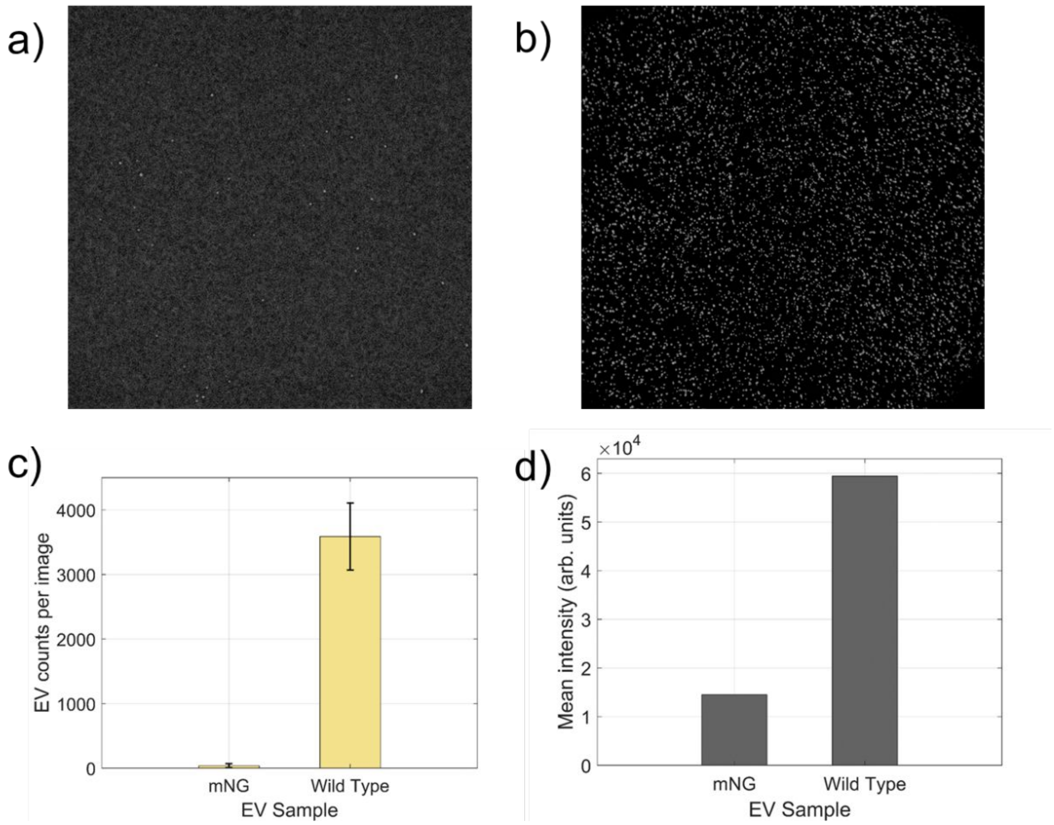

Figure S6. Representative fluorescence images of R-PE signals from a) anti-CD9-R-PE stained mNG-EVs and b) anti-CD9-R-PE stained wt-EVs, both with view size 133 μm × 133 μm. c) Averaged numbers of detected EVs per image for both samples. Twenty images were taken for both samples to obtain the average. d) Mean fluorescence intensities of both samples.

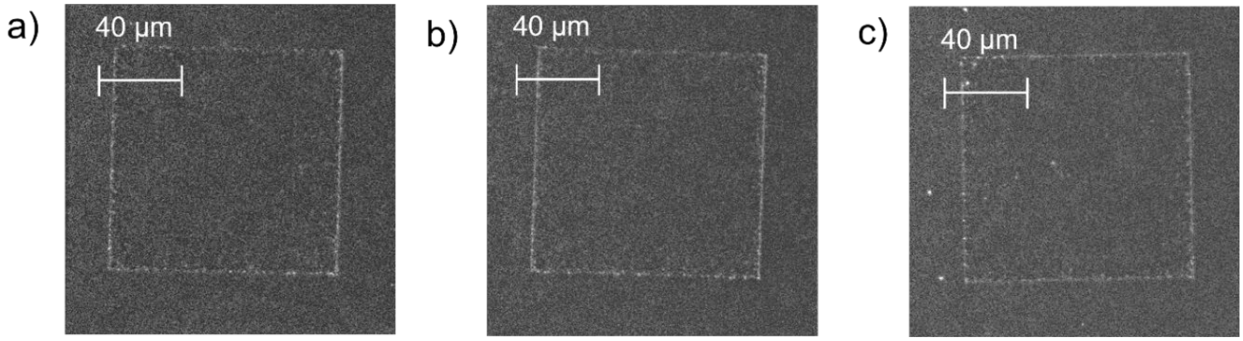

Figure S7. Fluorescence images of a) a nanohole array, b) the nanohole array after PLL functionalization, and c) the nanohole array after incubation with HEK293 EVs but without immunostaining as control.

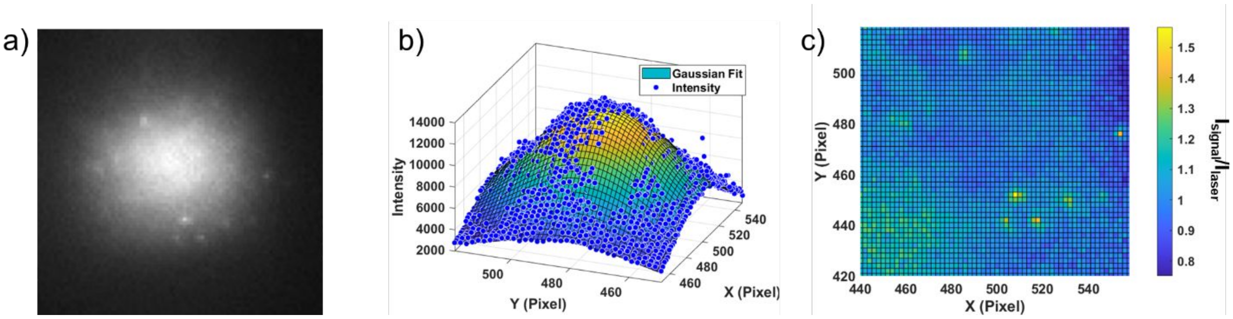

Figure S8. a) Bright-field fluorescence image of immunostained EVs in a nanohole array, view size 40 µm × 40 µm. b) Extracted intensity from the image with the laser intensity extracted using a Gaussian fit. c) Ratio of fluorescence spot intensity to extracted laser intensity.

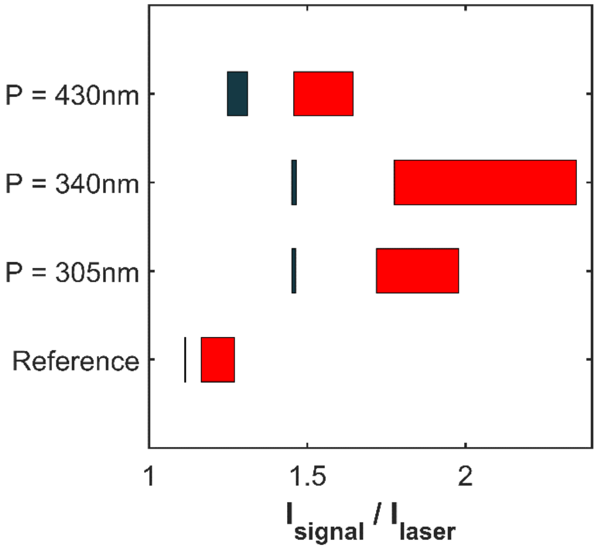

Figure S9. Top and bottom 10% of the fluorescence intensity distributions (I_{signal} / I_{laser}) shown as histograms in Figure 4b. The bottom 10% of the distribution expands by 11x, 13x, and 55x for the nanohole arrays with nanohole periods of 305 nm, 340 nm, and 430 nm, respectively, compared to the reference substrate. On the other hand, the top 10% of the distribution expands by 3x, 6x, and 2x as compared to the reference substrate. This implies that the higher number of EV counts observed in the case of the nanohole arrays is mostly due to the EVs with low fluorescence intensity that could be observed because of the fluorescence enhancement.

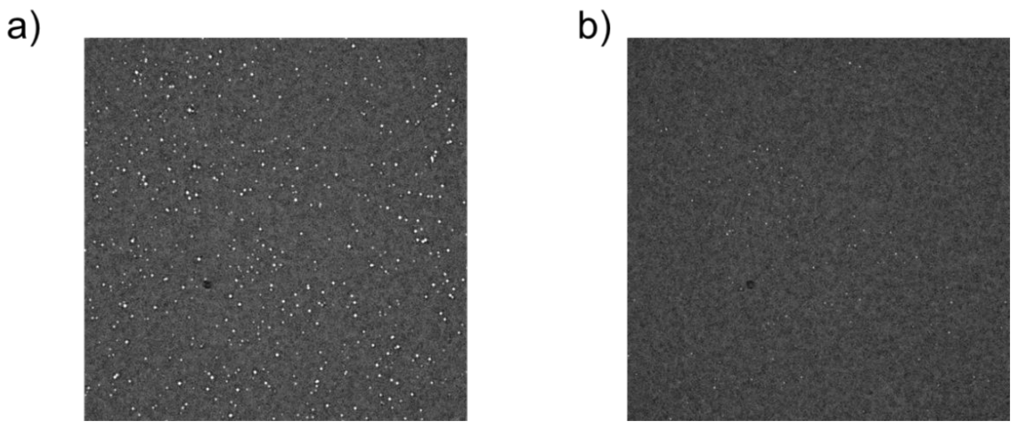

Figure S10. Wide-field fluorescence image of wt-EVs immunostained by R-PE tagged anti-CD9 lying on the top surface of (a) bare Al film and (b) bare glass substrate, both cases functionalized with PLL. Image view size: 133 µm x133 µm.

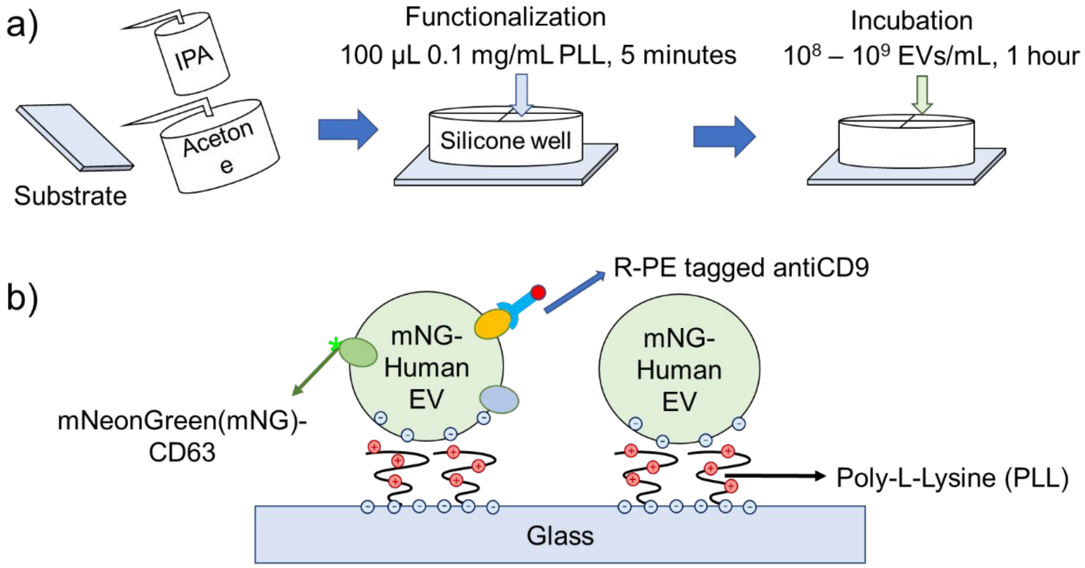

Figure S11. a) Schematic of the EV capturing procedure. The substrate was sequentially cleaned with isopropanol, acetone, and DI water. A silicone well was attached to the substrate for functionalization with poly-L-lysine (PLL). 0.1 mg/mL of aqueous PLL solution was inserted and left there for 5 minutes, after which the well was washed thoroughly with DI water. The mNG-EV solution was inserted and incubated for 1 hour for EV capturing. Lastly, the well was washed with plenty of PBS. b) Schematic of two EVs captured on a PLL functionalized glass substrate and immunostained by R-PE tagged anti-CD9.

Table S1. Comparison between this work and other similar works.

	Location of the detected EVs	Enhanced fluorescence intensity of fluorophores on EVs	Enhanced density of detected EVs	Reference
Gold nanohole arrays	Both on Au film and in nanoholes	8.6	10	[23]
Gold nanohole arrays	Both on Au film and in nanoholes	< 8	< 8	[18]
**Al nanohole arrays**	**Only in nanoholes**	**1.3**	**12**	**This work**
